# Effects of Tormentic Acid and the Extracts from *Callistemon citrinus* on the Production of Extracellular Proteases by *Staphylococcus aureus*

**DOI:** 10.1155/2020/6926320

**Published:** 2020-04-21

**Authors:** Rumbidzai Mashezha, Molly Mombeshora, Stanley Mukanganyama

**Affiliations:** Department of Biochemistry, University of Zimbabwe, Harare, Zimbabwe

## Abstract

*Staphylococcus aureus* is among the common nosocomial pathogens. Antibiotics have been used to treat *S. aureus* infections. However, there has been increased mortality associated with drug-resistant strains of *S. aureus*. Extracellular proteases have been implicated to be responsible for the transition of *S. aureus* from an adhesive pathogen to an invasive pathogen. The development of resistant strains has necessitated the search for new sources of drugs. Plants have been traditionally used as sources of therapeutic molecules. The objective of this study was to determine the effect of tormentic acid and the extracts from *Callistemon citrinus* on the production of extracellular proteases by *S. aureus*. The broth microdilution antibacterial susceptibility assay was used to determine the antibacterial effects of tormentic acid and the extracts on *S. aureus*. Both extracts showed a minimum inhibitory concentration (MIC) value of 50 *μ*g/ml. The water : ethanol (50 : 50) and the dichloromethane : methanol (50 : 50) extracts were found to be bactericidal against S. *aureus* at a concentration of 100 *μ*g/ml and 50 *μ*g/ml, respectively. The effect of tormentic acid and extracts on extracellular protease production was investigated using the protease assay. A zone of proteolytic activity (Pr) was measured as the ratio of the diameter of the colony to the total diameter of colony plus zone of hydrolysis. The extracts reduced the production of extracellular proteases, while tormentic acid completely inhibited the production of extracellular proteases by *S. aureus*. The Pr value for tormentic acid was found to be 1. The Pr values of the dichloromethane : methanol extract and the water : ethanol extract were 0.92 and 0.84, respectively. In conclusion, tormentic acid was shown to inhibit extracellular protease production; therefore, there is need to explore its use in antivirulence therapy to combat *S. aureus* infections.

## 1. Introduction

There has been an increase in severe *Staphylococcus aureus* infections caused by drug-resistant strains [1]. This increase in infections has been partly attributed to the hypersecretion of proteases by the bacteria [2]. Extracellular proteases are used by the bacteria to resist antibiotics, for survival during interaction with the innate immune system of host and formation of skin abscess and as effectors of virulence-determinant stability. Studies have shown that *S. aureus* strains which lack protease genes have decreased the abscess formation and impaired organ invasion [3]. Extracellular proteases are required for growth of bacteria in peptide-rich environments, serum, and blood and in the presence of antimicrobial peptides. Extracellular proteases increase the invasiveness of the bacteria into the body as they cleave proteins such as elastin. The cleaving of elastin allows the bacteria to enter into organs and the bloodstream allowing the bacteria to spread to different parts of the body. Extracellular proteases also assist in resisting phagocytosis by human leukocytes [4]. Proteases such as aureolysin have the ability to cleave the human cathelicin, therefore, conferring the bacteria's resistance to this protein [1].

The mechanism by which *S. aureus* cells control the production of virulence factors is through the action of secreted proteases [4]. Extracellular proteases are produced alongside toxins and other exoenzymes to control their stability [5]. The resulting stability facilitates the coordinated titration of virulence factors to suit niche specific situations. Therefore, extracellular proteases can be considered as the master regulators of virulence as they control the coordinated expression of other virulence factors [6]. The inhibition of extracellular protease production can result in the reduction of the expression of other virulence factors. *Callistemon citrinus* commonly known as bottlebrush because of its cylindrical brush like flowers is widely distributed in the temperate regions particularly in Australia, South America, and tropical Asia [7] and is exotic to Zimbabwe. *C. citrinus* belongs to the family *Myrtaceae*. Different parts of *C. citrinus* have been used in folk medicine to treat diarrhoea, dysentery, rheumatism, and bronchitis. Previous work carried out on *C*. *citrinus* resulted in the isolation and identification of three compounds, namely, tormentic acid, betulinic acid, and 23-hydroxyurs-12-en-24-oic. Tormentic acid was the most abundant isolated compound with a percentage yield of 1.3 % of the crude sample [8]. Tormentic acid is a triterpenoid which has been shown to have antimicrobial, antiatherogenic, anti-inflammatory, and antitumour activities [9]. Tormentic acid has also been shown to have hepatoprotective effects preventing liver damage caused by acetaminophen overdose. Tormentic acid inhibits the production of reactive oxygen species that is induced by acetaminophen overdose [10]. The study determined the antibacterial effects of tormentic acid and two extracts from *C*. *citrinus* against *S*. *aureus*. The aim of this study was to determine the effects of tormentic acid, isolated from *C*. *citrinus* in a previous study and two extracts from the same plant on the production of extracellular proteases by *S*. *aureus*.

## 2. Materials and Methods

### 2.1. Reagents and Chemicals

All chemicals used in this investigation were of analytical grade and were obtained from Sigma-Aldrich (Steinheit, Germany). These include dimethylsulfoxide (DMSO), ciprofloxacin, skimmed milk powder, 3-(4.5-dimethythiazol-2-yl)-2.5-diphenyl tetrazolium bromide (MTT), Luria broth base, and Luria Agar.

### 2.2. Microbial Strain and Culture Media

*S. aureus* ATCC 9144 was acquired from the Microbiological Section in the Department of Biological Sciences at the University of Botswana (Gaborone, Botswana). Bacteria were kept as glycerol stocks at −35°C. For each assay, bacteria were grown on a Luria agar for 24 h at 37°C, followed by inoculation in Luria broth. Inoculum concentration was adjusted to a concentration of 10^6^ c.f.u/ml using McFarland standard.

### 2.3. Collection and Preparation of Plant Material

*C*. *citrinus* leaves, voucher number UZ2 E7, were collected from Harare (University of Zimbabwe: 16.8^o^S, 31.1167^o^E). The leaf samples were authenticated by a taxonomist, Mr. Christopher Chapano of the National Herbarium and Botanic Gardens, Harare, Zimbabwe. The leaves were washed under running tap water to remove soil and dust particles and dried in an oven at 50°C. Preparation of plant extracts was carried out by first grinding the leaves to a fine powder using a mortar and pestle. Approximately 50 g powder was placed in a plastic beaker and 500 ml of 50 : 50 (v/v) DCM : methanol added to the powder. A second sample was prepared by placing 50 g powder in 500 ml of 50 : 50 (v/v) water : ethanol. The cold maceration method was used to extract phytochemicals from the powdered leaves. The mixture obtained was filtered through a No. 1 Whatman filter paper and concentrated under a vacuum using a rotary evaporator RII (BUCHI, LabortechnikAG, Switzerland). The extracts were dried to a constant mass under cold stream of air from fan in a fume hood cabinet. All extracts were stored in sterile centrifuge tubes at −4°C until use.

### 2.4. Screening for Antibacterial Activity

Minimum inhibitory concentrations (MICs) were determined using the (3-(4,5-dimethylthiazol-2-yl)-2,5-diphenyltetrazolium bromide MTT assay. The assay is a colorimetric method used to measure the activity of enzymes that reduce MTT to formazan, giving a purple colour in metabolically active cells [11]. The plant extracts were dissolved in DMSO final concentration of 2.5 % and diluted in Luria broth to required concentrations of 12.5, 25, 50, and 100 μg/ml into 96-well microtitre plates. Cultures of *S. aureus* (100 *μ*l) with a final concentration of 1 × 10^6^ c.f.u/ml were added to each well. Ciprofloxacin was used as the positive control, and Luria broth in 2.5% DMSO was included as a negative control. Microtitre plates were incubated for 24 hrs at 37° in a Lab Companion incubator (Jeio Tech. Co. Ltd, Seoul, Korea) under a closed humidified atmosphere. The growth of cells was quantified using a Genios Pro microplate reader (Tecan Group Ltd Mannedorf, Switzerland) using the change in optical density at 590 nm before and after 24 h incubation. As an indicator of growth, 25 *μ*l of 2 mg/ml MTT was added to each of the microtitre plate wells and incubated for 2 h. A purple colour was observed where there was growth of the bacteria, and a yellow colour was observed where there was no growth. The MIC was then determined as the lowest concentration that showed no growth. The minimum bactericidal concentration (MBC) was determined from the MIC plate. In order to determine the MBC, samples from the MIC microtitre plate were used. A loopful of inoculum was collected from the microtitre wells with concentrations less than or equal to the MIC. The samples were plated onto Luria agar in duplicate, and bacterial growth was evaluated after overnight incubation at 37°C for 24 h. The lowest concentration of the extract without growth was considered as the MBC.

### 2.5. Effect of Tormentic Acid and Extracts from *C. citrinus* on Extracellular Protease Production

The assay was performed based on the method reported by Vijayaraghavan and Vincent [12] with modifications. A volume of 400 ml of skimmed milk agar was prepared by dissolving meat extract 1 g/L, peptone 5.0 g/L, sodium chloride 8.0 g/L, agar 15 g/L, and skimmed milk 15 g/L. A mass of 0.006 g of bromocresol green dye was added. The solution was sterilised by autoclaving in an Accu Steriliser autoclave (VWR Scientific products Co., USA). A final concentration of 100 *µ*g/ml tormentic acid, 100 *µ*g/ml water : ethanol extract, and 50 *µ*g/ml DCM : methanol extract was added to separate centrifuge tubes containing 30 ml of media. Ciprofloxacin was used as the positive control at a concentration of 25 *µ*g/ml. For the negative control plate, 500 *µ*l of DMSO was added to the liquid agar. The agar was poured into six labelled Petri dishes, respectively, and allowed to solidify. *S. aureus* cells were diluted using Luria broth to a concentration of 1 × 10^8^ c.f.u/ml. A volume of 5 *µ*l of the cells was distributed onto the agar plates. The plates were incubated at 37°C for 48 hours. To determine the protease activity, the colony diameter and the zone of clearance around the bacterial colony were measured. The protease activity (Pr) was measured as the ratio of the diameter of the colony to the total diameter of colony plus zone of hydrolysis calculated as in the following equation:

(1) $$\begin{array}{c} \mathrm{Pr}=\frac{\text{colony diameter}}{\text{colony diameter }+\text{ zone of hydrolysis}}. \end{array}$$

## 3. Results and Discussion

### 3.1. Extract Preparation from *C. citrinus*

Two extracts were prepared from the leaves of *C*. *citrinus*. The water : ethanol extract had a higher percentage yield of 9.4 % compared with that of the DCM : methanol extract which had a yield of 1.6 %. The solvent mixture of water : ethanol possess the potential of extracting a wide range of phytochemicals [13]. Phytochemicals which can be extracted using this solvent mixture include flavonoids, sapononins, phenol hydroquinones, sterols, and alkaloids. In addition to this, water is known as a universal solvent because it dissolves a variety of different molecules. This is because water has the ability to form hydrogen bonds with other molecules. Water molecules have a polar arrangement on the oxygen and hydrogen atoms. On one side, hydrogen has a positive electrical charge, and on the other side, oxygen has a negative charge. This allows the water molecules to become attracted to many other different types of molecules [14].

### 3.2. Effects of Extracts and Tormentic Acid on the Growth of *S. aureus*

**Figure 1:**
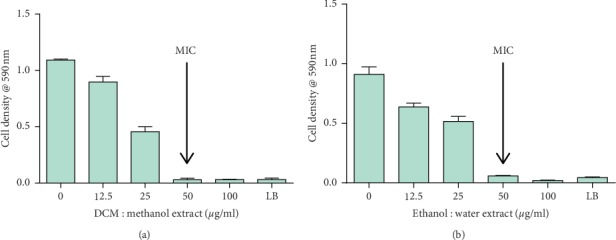
Effect of DCM : methanol extract and water : ethanol extract on *S. aureus*. Values are for mean ± standard deviation (error bar) for *n* = 4. The asterisks indicate a significant difference from the control with *p* < 0.05 and ^*∗∗∗∗*^*p* < 0.0001; ns means no significant difference.

The effects of the extracts from *C. citrinus* and tormentic acid on *S. aureus* were determined using the broth microdilution assay. Both extracts significantly inhibited the growth of *S. aureus* as shown in Figure 1.

The percentage inhibition of the DCM: methanol extract and the water: ethanol extract was 97.1% and 97.9%, respectively. The MICs of the two extracts were determined. Both extracts from *C. citrinus* were potent growth inhibitors of *S. aureus* with an MIC value of 50 *µ*g/ml. A concentration of 100 *µ*g/ml tormentic acid inhibited the growth of *S*. *aureus* by 72.7%. These findings are in agreement with those of the previous studies that show that tormentic acid exerts bacteriostatic effects against Gram-positive bacteria [15]. Ciprofloxacin, the positive control, inhibited the growth of *S. aureus*. The MIC of ciprofloxacin was found to be 0.125 *µ*g/ml. The MBC for both extracts was determined by plating *S. aureus* that was exposed to 25 *µ*g/ml, 50 *µ*g/ml, and 100 *µ*g/ml of each extract on Luria agar plates. Both extracts exhibited bactericidal activities against *S. aureus.* The MBC for the DCM: methanol extract was 50 *µ*g/ml while that for the water: ethanol extract was 100 *µ*g/ml. Phytochemicals or secondary metabolites present in *C. citrinus* which include terpenoids and alkaloids [16] may be responsible for the observed antibacterial activity. Phytochemicals exert their antibacterial activity through different mechanisms. For example, tannins act by iron deprivation or nonspecific interactions with vital proteins such as enzymes [17]. The most potent antibacterial phytochemicals present in *C. citrinus* leaves have been reported to be due to the presence alkaloids and phenols [18]. Based on the antibacterial activity of extracts from *C. citrinus* and tormentic acid observed in this study, both the extracts and tormentic acid were potent growth inhibitors. However, the extracts were more potent in inhibiting the growth of *S. aureus* compared with tormentic acid. Components in the extracts exhibited high antibacterial activities as compared with the isolated compound. This may be because, in extracts constituted of numerous compounds, and thus their antibacterial activity may involve synergistic interactions [19].

### 3.3. Protease Inhibitory Activity of Tormentic Acid and Extracts from *C. citrinus*

The protease assay was carried out to determine the effect of tormentic acid and extracts from *C. citrinus* on the production of extracellular proteases by *S. aureus*. The reference range of Pr values adapted from Elleboudy et al. [20] was used to interpret the proteolytic activity. The calculated Pr values for tormentic acid, extracts from *C*. *citrinus*, and control samples are as shown in Table 1.

Results in Table 1 show that no proteolytic activity was observed on the plate that contained tormentic acid. This indicates that the production of extracellular proteases was inhibited. Tormentic acid was able to inhibit the production of extracellular proteases by *S. aureus*; therefore, no proteases were available to be digested through the substrate in the nutrient agar. Therefore, tormentic acid completely inhibited the production of extracellular proteases by *S. aureus*. Tormentic acid is a triterpenoid, and these components have been shown to inhibit the growth of bacteria by reducing the production of secreted proteins such as *α*-toxin, staphyloccoccal enterotoxin A, and staphyloccoccal enterotoxin B [21]. The precise mechanism of protease inhibition by tormentic acid is not yet known. The computer modelling has proposed that triterpenes have the suitable molecular size fit into the hydrophobic interface site of the relaxed monomer, which inhibits the protease dimerisation [22]. Additional to the hydrophobic interactions, hydrogen-bonding is also suggested between protease and hydroxyl/carboxyl groups in triterpene scaffold [23]. Since the main objective of this study was to screen the inhibitory effect of tormentic acid and extracts from *C*. *citrinus* on protease production, further studies on other inhibition mechanisms using both biological assays and computer modelling need to be carried out in future studies. Weak and mild proteolytic activities were observed on the plates which contained the extracts from *C. citrinus*, while a strong proteolytic activity was observed on the negative control plate. The extracts reduced the amount of extracellular proteases produced by *S. aureus*. However, the zones of proteolysis around these colonies were relatively smaller compared with the zone of proteolysis on the negative control plate with cells only. The negative control plate had no extract, and a strong proteolytic activity was observed. This suggests that *S. aureus* produced extracellular proteases that digested the substrate in the nutrient agar resulting in a zone of proteolysis around the bacterial colony. For the water : ethanol extract, the proteolytic activity decreased as the extract concentration increased from 50 *µ*g/ml to 100 *µ*g/ml. Protease inhibition by plant extracts has been attributed to the inhibition of bacterial proteases involved in several physiological processes in addition to interactions between the inhibitor and the cell wall or proteins from the plasma membrane resulting in cell permeability changes and prompting the death of bacteria [24]. An agar plate containing ciprofloxacin was used as the positive control and showed no proteolytic activity by *S*. *aureus*. In bacteria virulence is controlled and coordinated by a process of intercellular communication known as quorum-sensing (QS). Several Gram-positive bacteria such as *S. aureus* use QS to coordinate the expression of production of virulence factors including the production of extracellular proteases. Studies have shown that some phytochemicals can act as quorum-sensing inhibitors [25]. The findings of this study suggest that the phytochemicals extracted from *C. citrinus* might have acted as inhibitors to reduce the production of extracellular proteases by *S. aureus*. At 100 *µ*g/ml, tormentic acid showed a partial inhibition of the growth (72.7 %) of *S*. *aureus* but managed to completely inhibit the production of proteases by the bacteria. Previous studies have shown that many antibacterial compounds that exhibit little influence on the overall growth of the bacteria can greatly affect the expression of S*. aureus* virulence factors [26]. Additional to targeting bacterial growth, potent therapeutic strategies should also employ antipathogenic or antivirulence therapies [27], which target cellular processes responsible for pathogenesis and virulence such as the production of extracellular proteases.

## 4. Conclusions

Extracts from *C. citrinus* and tormentic acid inhibited the growth of *S. aureus*. The extracts were bactericidal, while tormentic acid was bacteriostatic. The extracts also reduced the production of extracellular proteases by *S. aureus*, while tormentic acid completely inhibited the production of extracellular proteases by *S. aureus*. Tormentic acid inhibits the extracellular protease production and may be incorporated as antivirulence therapy to combat *S. aureus* infections.

## Figures and Tables

**Table 1:** Pr values obtained after exposing *S aureus* to tormentic acid and extracts from *C*. *citrinus*.

Sample	Pr value	Measure of protease production
Negative control	0.64	Very strong
Positive control	1	None
Tormentic acid (100 *µ*g/ml)	1	None
DCM : methanol extract (50 *µ*g/ml)	0.92	Weak
Water : ethanolic extract (100 *µ*g/ml)	0.92	Weak
Water : ethanol extract (50 *µ*g/ml)	0.85	Mild

Unexposed cells were used as the negative control and ciprofloxacin was used as the positive control. Protease activity was scored as follows: (1) No protease activity; (0.99–0.9) weak protease activity; (0.89–0.8) mild protease activity; (0.79–0.7) moderately strong protease activity; (<0.69) very strong protease activity.

## Data Availability

The data sets generated during and analysed during the current study are available from the corresponding author on reasonable request.

## Supplementary Materials

Supplementary Materials Tormentic acid used in this study was obtained by column chromatography from leaf extracts of *C. citrinus* in a previous study in our laboratory [8]. The chemical structure, formula, and data from mass spectrometry for the fractions obtained from column chromatography.
