# Supplementary material for: Effects of Tormentic Acid and the Extracts from Callistemon citrinus on the Production of Extracellular Proteases by Staphylococcus aureus
Source: Biochem Res Int. 2020 Apr 21;2020:6926320. doi: 10.1155/2020/6926320 (PMC7211245; doi:10.1155/2020/6926320)
Supplement: Supplementary Materials — Tormentic acid used in this study was obtained by column chromatography from leaf extracts of C. citrinus in a previous study in our laboratory [8]. The chemical structure, formula, and data from mass spectrometry for the fractions obtained from column chromatography. [file 6926320.f1.pdf]

Supplementary Material

Tormentic acid used in this study was obtained by column chromatography from leaf extracts of *C. citrinus* in a previous study in our laboratory [8]. The chemical structure, formula and data from mass spectrometry for the fractions obtained from column chromatography are shown in Table 1.

Table 1: Identity of fractions obtained from *C. citrinus* obtained from column chromatography

Fraction	Yield (g)	Identity	m/z	Theoretical Mass	Chemical Formula
**D1 181-183**	0.0148	Betulinic acid	456.3619 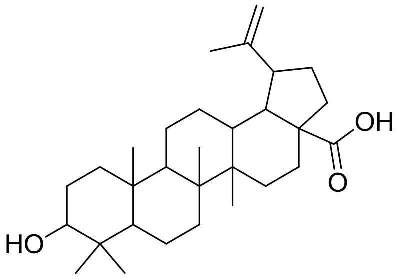	456.3603	$C_{30}H_{48}O_3$
**H1 200-214**	0.4484	Betulinic acid	456.3617	456.3603	$C_{30}H_{48}O_3$
**G2 259**	0.0227	Betulinic acid	456.3617	456.3603	$C_{30}H_{48}O_3$
**B1 163-169**	0.0089	Betulinic acid	456.3617	456.3603	$C_{30}H_{48}O_3$
**CC 190-192**	0.0212	Betulinic acid	456.3611	456.3603	$C_{30}H_{48}O_3$
**R2 335-339**	0.1196	Tormentic acid	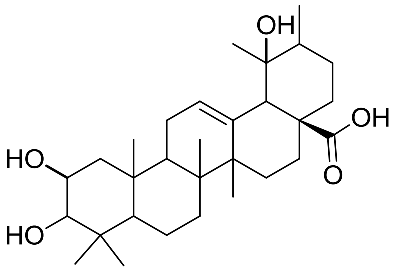	488.3502	$C_{30}H_{48}O_5$
**O2 317-325**	0.0297	Tormentic acid	488.3502	488.3502	$C_{30}H_{48}O_5$
**J2 282-284**	0.0380	Tormentic acid	488.3502	456.3603	$C_{30}H_{48}O_3$

**P2 326-330**	0.0297	Tormentic acid		488.3502	$C_{30}H_{48}O_5$
**K2 285-287**	0.1422	Tormentic acid		488.3502	$C_{30}H_{48}O_5$
**L2 290-294**	0.1557	Tormentic acid		488.3502	$C_{30}H_{48}O_5$
**M2 295-307**	0.4117	Tormentic acid		488.3502	$C_{30}H_{48}O_5$
**N2 308-316**	0.3797	Tormentic acid		488.3502	$C_{30}H_{48}O_5$
**I2 274-281**	0.2281	23-hydroxyurs-12-en-24-oic	472.9872 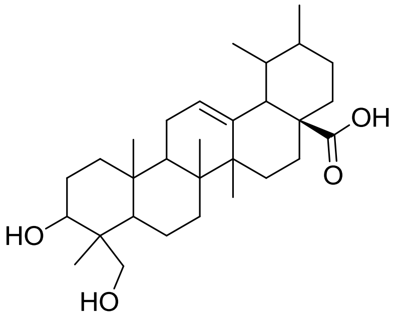	472.6997	$C_{30}H_{48}O_4$

A total mass of 100 g leaf extract of *C. citrinus* was loaded on silica gel. The column was developed using a gradient of 100 % hexane to 100 % ethyl acetate and then finally 10 % methanol. A total of 469 fractions were obtained. Fractions were typically labelled as: fraction 181-183 (D1) identified as betulinic acid, fractions 335-339 (R2) identified as tormentic acid and fraction 274-281 (I2) identified as 23-hydroxyurs-12-en-24-oic using mass spectrometry and nuclear magnetic resonance.

Data from Table 1 indicates that from the isolated and identified compounds, tormentic acid was the most abundant with a total yield of 1.3 % of the loaded sample.

Spectral data for tormentic acid obtained by nuclear magnetic resonance:

white powder; ^{1}H-NMR (DMSO, 400 MHz) δ (ppm): 5.14 (1H, *br* s, H-12), 3.49 (1H, m), 2.74 (1H, d, J = 9.2, H-3), 2.12 (1H, d, J = 11.2, H-18), 2.00-1.20 (CH_2 and CH region), 1.04 (3H, s, H-27), 0.93 (3H, s, H-23), 0.93 (3H, s, H-25), 0.92 (3H, s, H-30), 0.83 (3H, d, J = 6.4, H-29), 0.77 (1H, t, H-5), 0.75 (3H, s, H-26), 0.72 (3H, s, H-24). ^{13}C-NMR (DMSO, 100 MHz) δ (ppm); 178.7 (C-28), 138.7 (C-13), 124.9 (C-12), 82.7 (C-3), 70.2 (C-20), 67.6 (C-2), 55.2 (C-5), 52.8 (C-18) ,47.5 (C-17), 47.5 (C-1), 47.4 (C-9), 46.4 (C-14), 40.5 (C-10), 39.4 (C-8), 39.3 (C-4), 38.9 (C-21), 38.8 (C-19), 36.8 (C-22), 33.0 (C-7), 30.6 (C-16), 29.3 (C-23), 27.9 (C-15), 23.7 (C-27), 23.4 (C-11), 21.5 (C-30), 18.6 (C-6), 17.6 (C-24), 17.5 (C-26), 17.4 (C-24) , 16.9 (C-29), 16.9 (C-25).

Spectra:

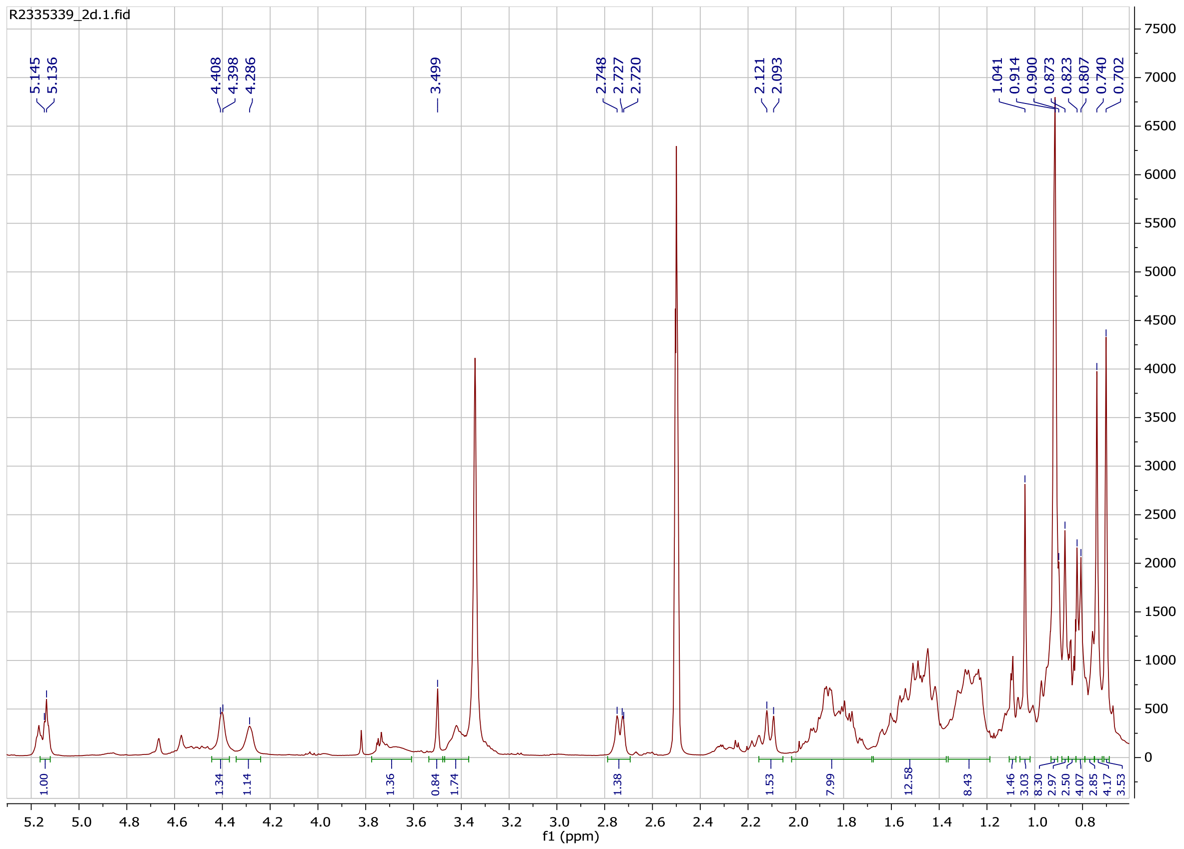

^{1}H NMR (DMSO, 400 MHz) Spectrum of R2 335-339.

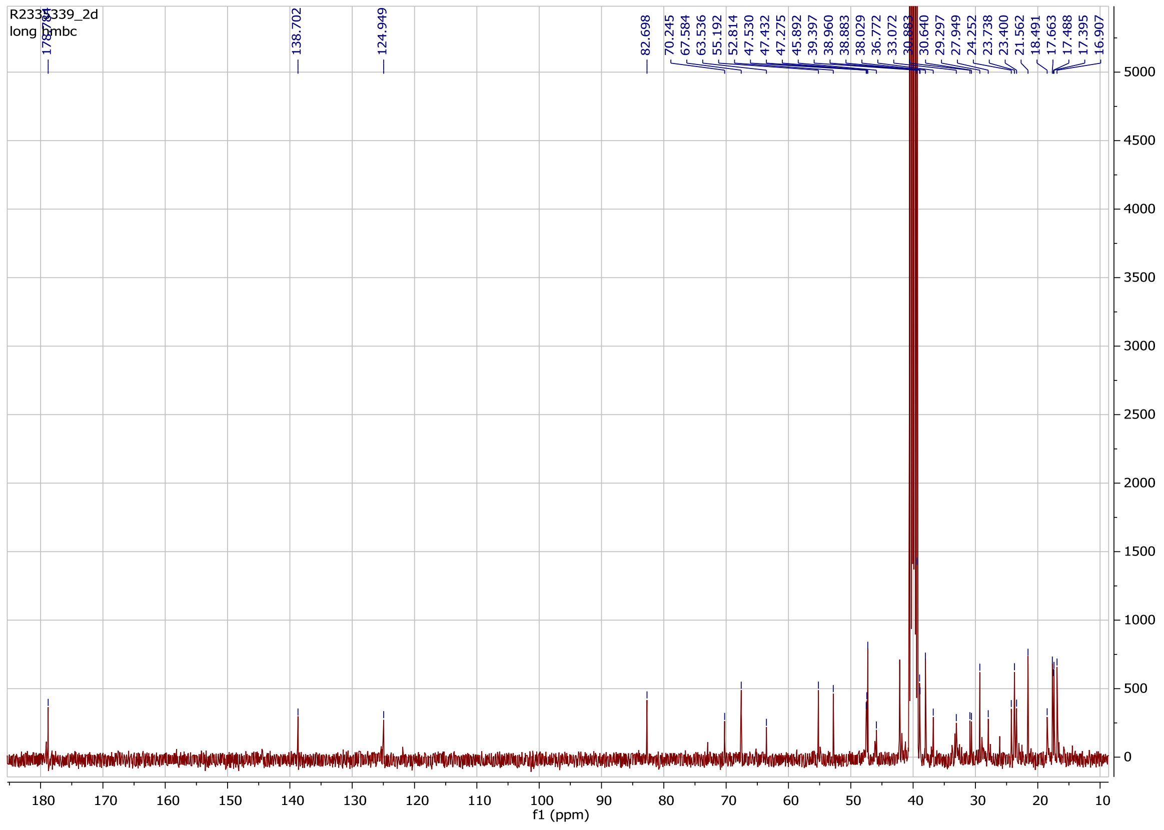

^{13}C NMR (DMSO, 100 MHz) Spectrum for R2 335-339.

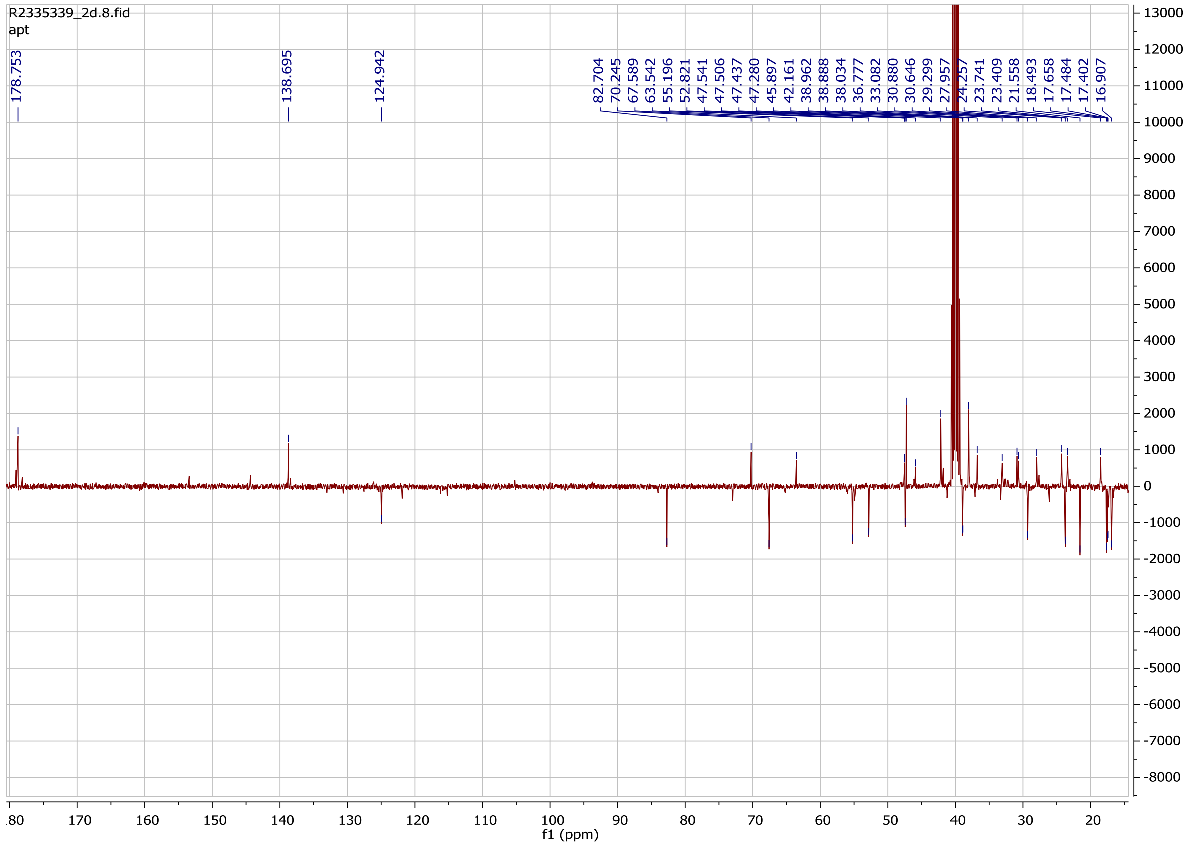

^{13}C APT-NMR (DMSO, 100 MHz) Spectrum for R2 335-339.
